# Leukocytoclastic Vasculitis with Systemic Involvement Associated with Ciprofloxacin Therapy: Case Report and Review of the Literature

**DOI:** 10.7759/cureus.900

**Published:** 2016-11-28

**Authors:** Bruno Morgado, Catarina Madeira, Joana Pinto, Joana Pestana

**Affiliations:** 1 Department of Biomedical Sciences and Medicine, University of Algarve; 2 Department of Internal Medicine, Hospital Centre of Algarve

**Keywords:** ciprofloxacin, vasculitis

## Abstract

A 71-year-old woman presented with constitutional signs and lower extremity palpable purpura after being prescribed a four-day course of 500 mg of ciprofloxacin two times daily for a gastrointestinal infection. She was admitted for inpatient treatment. During the third hospital day, she presented with an episode of abundant hematemesis while her skin lesions remained unchanged. Upper endoscopy revealed multiple lesions consistent with vasculitis and histological examination of the skin biopsy disclosed a leukocytoclastic vasculitis. The patient was successfully treated with prednisone following ciprofloxacin discontinuation. Complete resolution of the lesions on drug withdrawal strongly suggested drug toxicity, which was further supported by a score of 8 in the Naranjo Adverse Drug Reaction Probability Scale. Awareness that the development of skin and gastrointestinal lesions following administration of ciprofloxacin may be a manifestation of ciprofloxacin-induced vasculitis can help early detection, treatment, and lead to an overall good prognosis.

## Introduction and background

Ciprofloxacin is a generally safe and extensively used drug in everyday clinical practice only rarely associated with hypersensitivity reactions. These reactions are largely characterized by pruritus, rash, or photosensitivity and occur in less than 2% of users [1].

We described a case of an elderly woman with several comorbidities that developed leukocytoclastic vasculitis (LCV) with cutaneous and gastrointestinal involvement after a four-day course of ciprofloxacin for the treatment of a gastrointestinal infection. The authors consider relevant to report this case because, although ciprofloxacin-related cutaneous vasculitis is rare, the diagnosis can be challenging as the clinical picture may be indistinguishable from other forms of vasculitis. The diagnosis requires a high degree of suspicion, particularly due to a possibly fatal outcome. It is important for clinicians to be alerted to this potential adverse effect that can be easily treated with drug discontinuation. Additionally, the case presents gastrointestinal involvement, which had been unreported to date, and was successfully treated with drug withdrawal and glucocorticoid therapy.

## Review

### Case report

**Figure 1:**
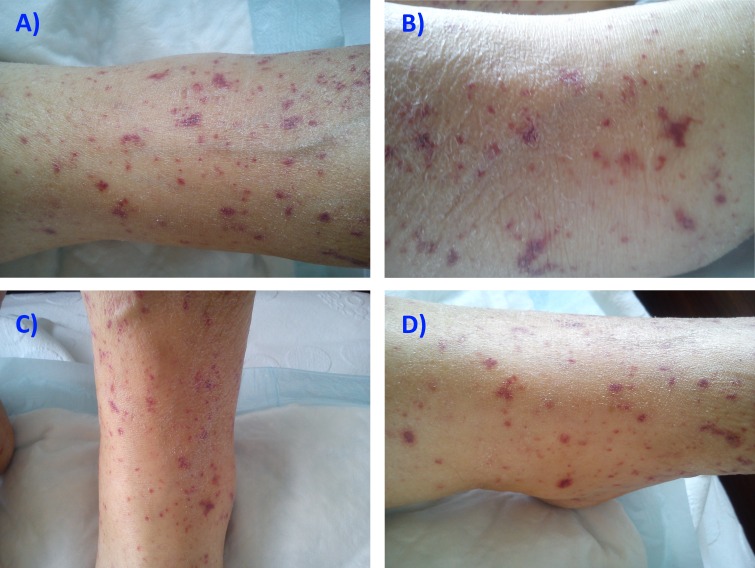
Macroscopic view of the main lesions on admission The lesions cover the anterior and medial aspect of the lower limbs. They are violaceous discolorations of heterogeneous asymmetric shapes, sharp borders, and of various sizes ranging from 1 to 6 mm.

A 71-year-old overweight woman with no personal or family history of dermatologic conditions, atopy, or adverse drug reactions presented to her primary care facility with a 12-hour history of abundant watery diarrhea, together with colic-type abdominal pain, vomiting, and negative for stool blood, pus, or mucus. Her history was positive for essential hypertension. She was observed by her primary care provider and was prescribed ciprofloxacin and an oral saline rehydration formula after which she was discharged. Four days later, she presented to our emergency department with no symptomatic improvement and complained of new onset arthralgias, myalgias, fatigue, and lower limb skin lesions described as small, reddish, slightly pruriginous macules. Her medications included telmisartan, 40 mg daily, hydrochlorothiazide, 12.5 mg daily, and the recently prescribed ciprofloxacin, 500 mg two times daily. She referred to taking paracetamol, 1 g once to twice a week, for an occasional headache and denied use of any other over-the-counter or herbal medications. Physical examination was remarkable for signs of dehydration, a tympanic temperature of 38.3°C, blood pressure of 107/54 mmHg, pulse of 87/min, respiratory rate of 19/min, and pulse oximetry of 98% on ambient air. Abdominal examination revealed only a slightly tender abdomen on palpation. Neurologic examination was normal. The skin presented with small, multiple violaceous discolorations of various sizes, ranging from approximately 1 to 6 mm, visible over the medial and anterior aspect of the lower part of both lower limbs (Figure 1A-1D). Informed patient consent was obtained at the time of treatment.

The lesions were non-blanching, tender, and slightly raised on palpation. Femoral and distal pulses were present, and there was no palpable femoral or inguinal lymphadenopathy as well as any further dermatological findings. Laboratory analysis disclosed a slight leukocytosis (12.3 x 10 9/L) and a C-reactive protein elevation (63 mg/L), hyperkalaemia (5.65 mmol/L), hypernatremia (156 mmol/L), elevated blood urea (30 mg/dL), and serum creatinine (1.2 mg/dL). She was admitted on the pretext of dehydration with inability to tolerate oral food or fluid intake, and the following acute problems were considered:

- Acute gastroenteritis of probable viral aetiology - Renal impairment of mixed, but mainly pre-renal aetiology - Electrolytic abnormalities - Violaceous lesions on lower limbs of unknown aetiology

Oral and intravenous rehydration were undertaken as needed, and ciprofloxacin was not discontinued as other causes for the presented clinical findings were still being considered.

The day after admission, a skin punch biopsy was performed. In the search for evidence of systemic disease, further investigations were conducted that revealed no abnormalities in sedimentation rate, serum complement proteins C3 and C4, immunoglobulin A (IgA), angiotensin-converting enzyme (ACE), and circulating immunocomplexes. Anti-nuclear antibodies, antineutrophil cytoplasmic antibody (ANCA), anti-histone antibodies, rheumatoid factor, and anti-streptolysin-O were also negative. Serology for hepatitis B and C, as well as HIV, was negative. There was no indication of eosinophilia. After intravenous rehydration therapy, renal function and serum electrolytes improved and returned to baseline over the following days.

**Figure 2:**
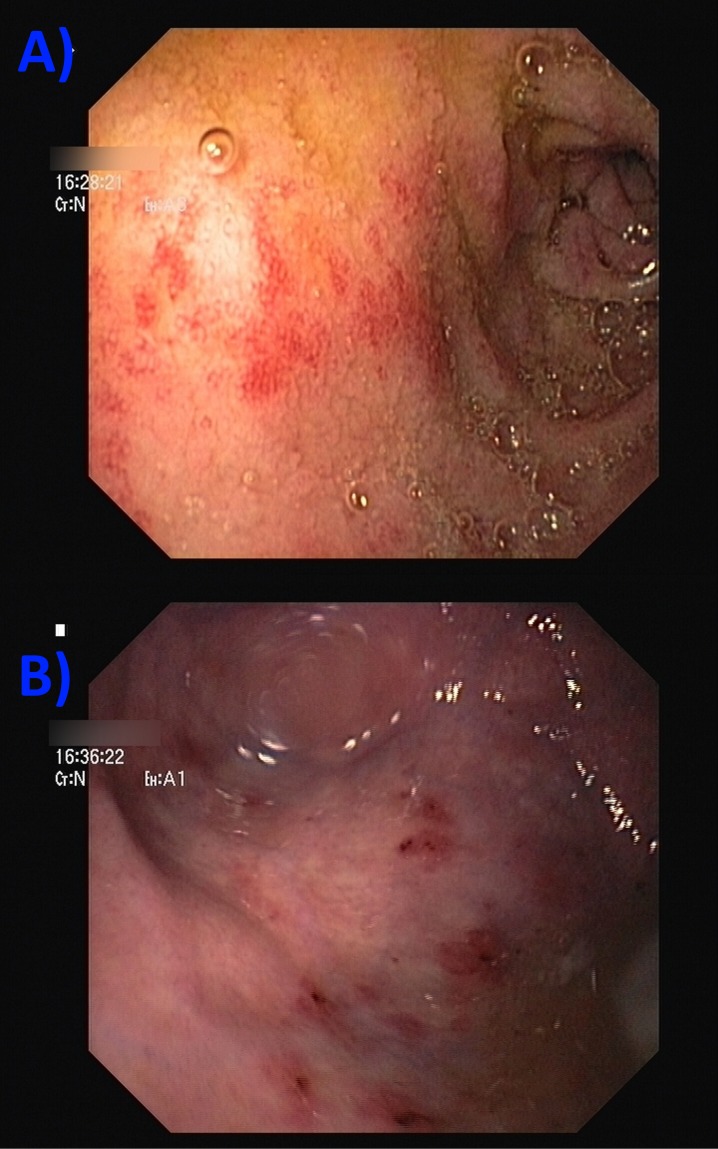
Endoscopic appearance of the gastric lumen Lesions are observable over the (A) duodenal bulb and (B) gastric fundus, both consistent with vasculitis.

During the third hospital day, the pruritic lesions showed no signs of improvement and the patient presented with the sudden onset of hematemesis. Upper endoscopy revealed the presence of violaceous lesions of variable sizes over the gastric fundus and duodenal bulb, similar to the presenting skin lesions, and consistent with vasculitis (Figure 2A-2B).

**Figure 3:**
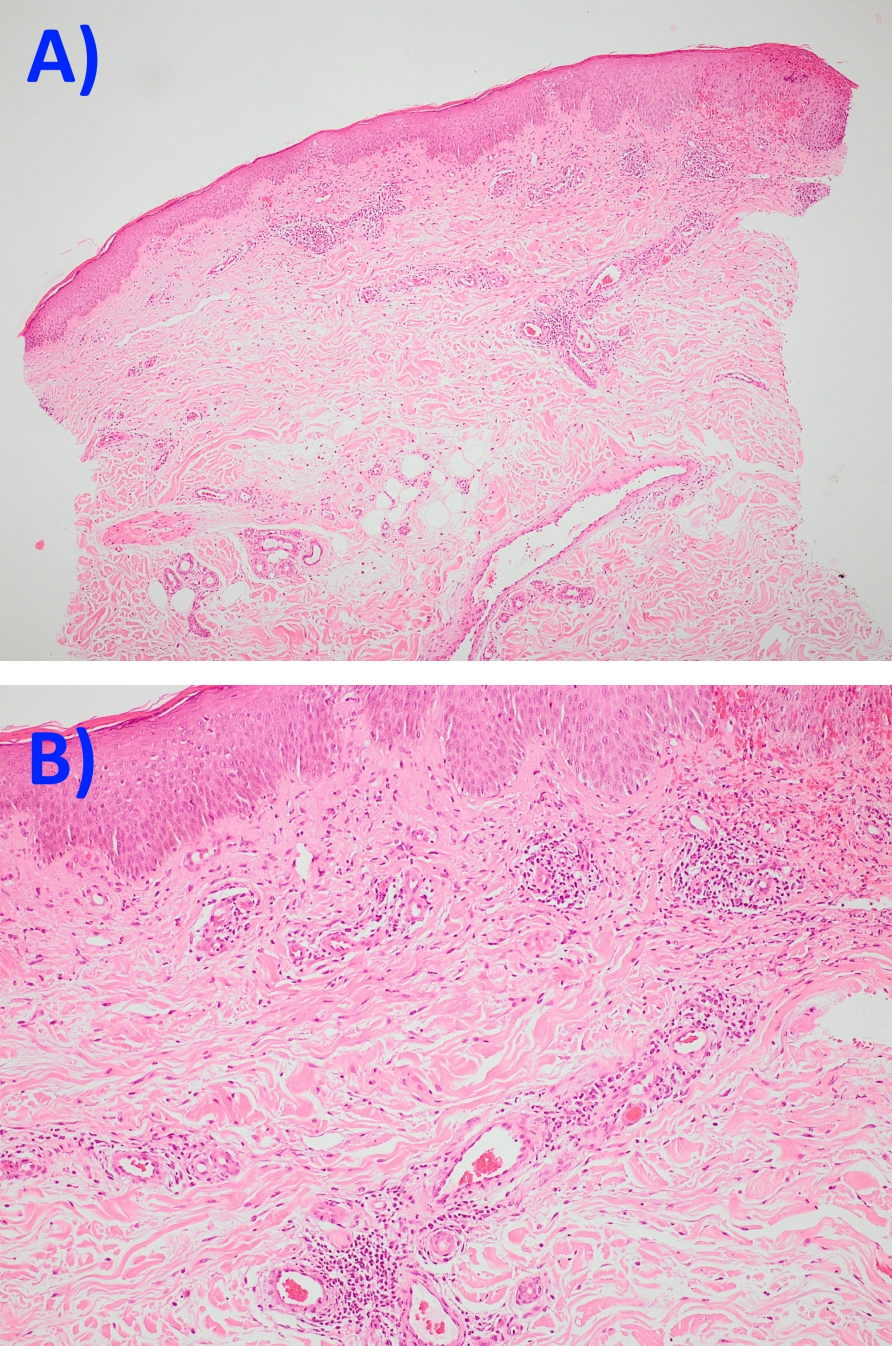
Microscopic view of the skin lesions Leukocytoclastic vasculitis with fragmented neutrophils, red blood cell extravasation, and mild fibrinoid necrosis of the vascular wall.

Biopsies were not performed so as to avoid inducing further blood loss. Upper endoscopy also revealed what seemed to have been a significant amount of blood originating from the airway. Lower endoscopy was also performed and revealed no significant findings. Given the upper endoscopic findings of blood originating from the airway, plain chest X-ray and high-resolution computer tomography (CT) were ordered for the suspected respiratory system involvement and revealed no discernible abnormalities. The patient refused bronchoscopy examination. Urinalysis and blood culture results were normal, and the rest of the physical examination showed no evidence of vasculitis involving other organ systems. At this time, the suspicion arose of systemic vasculitis associated with ciprofloxacin. This was followed by the immediate discontinuation of the drug. She was given a course of prednisone at a dose of 0.5 mg/kg daily with gradual improvement of the skin lesions over the following week and no new episodes of hematemesis. Biopsy of the skin lesions showed areas of LCV with a fragmentation of neutrophils and red blood cell extravasation. Other areas displayed features of oedema of the vessel wall and inflammatory infiltration with fibrinoid necrosis (Figure 3A-3B).

We assumed the presence of systemic vasculitis, and no further invasive diagnostic procedures were performed. We decided to further validate the diagnosis by resorting to the Naranjo Adverse Drug Reaction (ADR) Probability Scale, commonly used in clinical practice to determine the likelihood of whether an adverse drug reaction is due to a suspected drug or other causes (Table 1) [2].

**Table 1:** Naranjo Adverse Drug Reaction (ADR) Probability Scale Scoring ≥ 9 = definite ADR; 5 - 8 = probable ADR; 1 - 4 = possible ADR; 0 = doubtful ADR.

Sl No		Yes	No	Do not know	Score in this case
1	Are there previous conclusive reports on this reaction?	+1	0	0	+1
2	Did the adverse event occur after the suspected drug was administered?	+2	-1	0	+2
3	Did the adverse reaction improve when the drug was discontinued or a specific antagonist was administered?	+1	0	0	+1
4	Did the adverse reaction reappear when the drug was re-administered?	+2	-1	0	0
5	Are there alternative causes (other than the drug) that could have, on their own, caused the reaction?	-1	+2	0	+2
6	Did the reaction reappear when a placebo was given?	-1	+1	0	0
7	Was the drug detected in the blood (or other fluids) in concentrations known to be toxic?	+1	0	0	0
8	Was the reaction more severe when the dose was increased or less severe when the dose was decreased?	+1	0	0	+1
9	Did the patient have a similar reaction to the same or similar drugs in any previous exposure?	+1	0	0	0
10	Was the adverse event confirmed by any objective evidence?	+1	0	0	+1
				Total	8

In our case, the probability of a drug-induced LCV adverse reaction is scored as “probable” (score 8, with a higher than 8 required for a definitive diagnosis). Given the severity of the previous clinical picture, we did not consider the possibility of reexposing the patient to the drug, a key point in many algorithms for a definitive diagnosis but seldom performed in clinical practice. Thus, by excluding other causes of systemic and gastrointestinal disease, we concluded that the most probable diagnostic hypothesis in the present case was a ciprofloxacin-induced cutaneous vasculitis with gastric involvement.

### Outcome and follow-up

The patient made a good clinical recovery with complete resolution of diarrhoea and restoration of normal renal function. Upon discontinuation of the ciprofloxacin, the violaceous lesions displayed significant progress; within 48 hours, most lesions showed signs of improvement. There was complete resolution of the skin and gastrointestinal lesions on the upper endoscopy at 11 days after admission. The patient was discharged without symptoms and with almost complete resolution of the skin findings 12 days following admission. The adverse drug reaction was recorded as a known condition in the patient’s medical record, and assistant physicians were notified of the present hypersensitivity and the possibility of cross-reaction with other agents in the same drug class. At follow-up consultations two, six, and 12 months after discharge, she remained clinically well with all observations within the normal range.

### Discussion

Following evaluation of the patient’s clinical history, laboratory and physical examination findings, a drug-induced cutaneous vasculitis was suspected with ciprofloxacin as the culprit. The time between first exposure to the drug and the onset of the first clinical findings range from three days to five weeks. This reaction appears to be reported in first-time users of the drug as well as with patients who took ciprofloxacin beforehand with no history of drug-induced vasculitis [3-12].

LCV is an uncommon but potentially serious side effect of ciprofloxacin. It is a small-vessel vasculitis characterized histologically by leukocytoclasis and presenting cardinal features, such as palpable, violaceous papules, affecting most commonly the lower part of the legs. Other manifestations, such as fever, arthralgias, lymphadenopathy, and an elevated erythrocyte sedimentation rate, are present in most patients [13-14]. The exact mechanism of LCV remains unknown but is thought to be the result of a type III hypersensitivity reaction with deposition of immune complexes and consequent damage to blood vessels by neutrophils [15]. Schmid, et al. mentioned that quinolones can cause hypersensitivity reactions through several different pathophysiological mechanisms [16]. The clarification of such mechanisms may change our understanding of how to prevent this reaction in some patients.

There is a paucity of reports in the literature regarding cutaneous LCV with or without systemic involvement association with ciprofloxacin therapy. From 1989 to date, we found only 13 case reports of LCV related to ciprofloxacin use (Table 2) [3-12].

**Table 2:** Prior Case Reports of Ciprofloxacin-induced Vasculitis LCV: leukocytoclastic vasculitis

Dose	Time to clinical onset	Other medications	Diagnostic method	Affected organ-systems	Final diagnosis	Treatment	Year published
500 mg po BID	3 days	None	Skin biopsy	Cutaneous	Mononuclear-cell infiltrate	Removal of ciprofloxacin	1989
500 mg po QD	4 days	Cephradine	Skin biopsy	Cutaneous	LCV	Removal of ciprofloxacin	1992
Unknown	10 days	Diuretic	Clinical	Cutaneous	Haemorrhagic vasculitis	None	1992
Unknown	4 days	Unknown	Skin biopsy	Cutaneous	Mononuclear-cell infiltrate	Removal of ciprofloxacin	1994
500 mg po BID	4 days	Fluoxetine	Clinical	Cutaneous	Cutaneous vasculitis	Removal of ciprofloxacin	1997
375 mg	Unknown	Ceftriaxone	Skin biopsy and rechallenge test	Cutaneous	LCV	Removal of drug and negative ceftriaxone challenge test	1997
Unknown	Unknown	Oral antidiabetic, nifedipine and digoxin	Clinical and skin biopsy	Cutaneous and renal vasculitis	LCV	Removal of drug and prednisone treatment	2001
Unknown	3 days	Several drugs	Skin biopsy	Cutaneous	LCV	Removal of ciprofloxacin	2004
Unknown	10 days	None	Clinical and biopsy	Cutaneous and renal vasculitis	Cutaneous and renal vasculitis	Removal of drug and prednisone treatment	2007
Unknown	7 days	Rifampin	Clinical	Cutaneous	Drug-induced vasculitis	Removal of ciprofloxacin	2009
Unknown	10 days	Flucloxacillin	Clinical	Cutaneous	Drug hypersensitivity	Removal of ciprofloxacin	2009
500 mg po BID	4 days	Clindamycin	Clinical	Cutaneous	Haemorrhagic vasculitis	Removal of ciprofloxacin	2010
400 mg IV BID	6 days	Clindamycin	Clinical	Cutaneous	Haemorrhagic vasculitis	Removal of ciprofloxacin	2010

They described patients with a variety of clinical presentations, mostly presenting cutaneous LCV and constitutional signs, and a few provided confirmation of additional organ system damage. Of these, two cases reported LCV with renal involvement and only Storsley, et al. confirmed renal vasculitis with biopsy [7, 9]. To the extent of our knowledge, the literature does not mention any case of ciprofloxacin-related vasculitis with gastrointestinal involvement [3-12]. Even though renal vasculitis with ciprofloxacin-induced LCV is rare, and gastrointestinal involvement has not been documented, drug-induced LCV is known to involve several systems, including the lungs and gastrointestinal tract [17-21]. Pace, et al. described systemic vasculitis with cutaneous, gastric, and renal involvement with the use of ofloxacin, another quinolone [22]. Likewise, Ceyhan, et al. reported an ofloxacin-induced vasculitis affecting the stomach and colon that presented clinically with bloody diarrhoea [23]. This is consistent with our patient presenting with hematemesis and endoscopic findings consistent with vasculitis following seven days of treatment. Not performing a gastric biopsy prevents us from making an accurate diagnosis of gastric involvement in our patient. Nevertheless, based on the clinical evidence of hematemesis, skin biopsy, and upper endoscopic findings before and after drug removal, we are confident that the gastric and duodenal mucosa presented with histologically similar lesions to the ones found on the skin, all attributable to treatment with ciprofloxacin.

Every patient believed to suffer from cutaneous vasculitis should undergo a detailed history, physical, and laboratory assessment. Diagnosing cutaneous vasculitis requires determination of the calibre of the vessels involved. Punch biopsy should be the method of choice since, as mentioned, LCV affects small vessels and the nature of the inflammatory infiltrate (neutrophilic, lymphocytic, or granulomatous) is one of the main factors considered for diagnosis. Since the composition of the infiltrate changes rapidly, an early biopsy is best if a reliable diagnosis is to be made [24]. Establishing the relationship between the offending agent and LCV is accomplished clinically and by the evident relationship between drug exposure and clinical presentation [25]. Some laboratory findings, such as ANCA and anti-histone antibodies, are occasionally helpful in the diagnosis of drug-induced vasculitis [9].

Cross-reactivity for the development of maculopapular exanthema with one fluoroquinolone predicts a recurrence with another fluoroquinolone for a small percentage of patients. Ball, et al. predicted a 10.4% probability of rash recurrence when taking ciprofloxacin in women who previously developed an exanthema with gemifloxacin. In the same study, several female patients who initially tolerated ciprofloxacin developed a rash when the treatment was again repeated with the same drug [26]. Even though cross-reactivity between ciprofloxacin and other quinolones have been described with the development of delayed-onset maculopapular cutaneous eruptions, to our knowledge, only one case has suggested an LCV resultant from cross-reactivity between quinolones. Blyth, et al. reported an LCV related with levofloxacin in a patient previously exposed to ciprofloxacin, raising the possibility of cross-reactivity reactions between quinolones, which can extend to full-blown LCV [24].

Owing to the rare nature of the phenomenon, no specific directives exist in managing ciprofloxacin-induced systemic LCV. Martinez-Taboada, et al. stated that discontinuation of the offending agent usually leads to resolution of the signs and symptoms in drug-induced LCV within days to weeks without the need of additional treatment. Some patients with joint symptoms may require non-steroidal anti-inflammatory drugs. Patients with a chronic or systemic disease may benefit from prednisone therapy. A minority of patients has been reported to require the addition of immunosuppressive agents, such as azathioprine or cyclophosphamide, to prednisone [27]. Most patients with this condition have a benevolent course following drug withdrawal, which should usually allow physicians to avoid overtreating these patients. Nevertheless, Pace, et al. described a case of fatal vasculitis associated with ofloxacin, and several reports have been made for drug-induced vasculitis with poor outcomes [18, 22]. In view of the knowledge currently available, and considering the age, comorbidities, and systemic involvement which was particular to our patient, pre-emptive glucocorticoid therapy was deemed appropriate for 10 days. This proved to be adequate and consistent with other cases, as treatment duration for all patients in the reported cases ranged from four to 10 days [3-12].

Even though the safety profile of quinolones is similar to other classes of antibacterial agents, severe low-frequency adverse drug hypersensitivity reactions, which may include vasculitis, have occurred with a small number of specific quinolones, namely, ciprofloxacin, ofloxacin, and levofloxacin [24]. Ciprofloxacin is overall a well-tolerated and widely used drug that has been available for more than 25 years for the treatment of a multitude of bacterial infections [1]. The most common adverse effects reported involve the gastrointestinal tract, with less than 8% of patients experiencing side effects, such as nausea, vomiting, and diarrhoea. Central nervous system involvement is less common, with less than 6.4% of users enduring effects, such as headaches and dizziness. Only as few as 3.6% of patients go on to experience phototoxicity and hypersensitivity reactions in which vasculitis is included [28].

## Conclusions

Consistent with preceding case reports linking the use of ciprofloxacin to LCV, we consider the former drug as the probable cause of LCV in our patient. Our patient presented with systemic vasculitis that included cutaneous and gastrointestinal involvement, which is unreported to date. Review of the current literature suggests the possibility of a cross-reaction between quinolones in causing LCV. As with previous case reports, our patient made a full recovery with prednisone treatment and removal of ciprofloxacin. While this condition is generally benign, it is important to recognize drug-induced LCV as it can be fatal without timely drug withdrawal.
